# Supplementary material for: Cellular and Molecular Mechanisms and Effects of Berberine on Obesity-Induced Inflammation
Source: Biomedicines. 2022 Jul 19;10(7):1739. doi: 10.3390/biomedicines10071739 (PMC9312506; doi:10.3390/biomedicines10071739)
Supplement: Supplementary file 1 [file biomedicines-10-01739-s001.zip › biomedicines-1803739-Supplementary.pdf]

Supplementary Figures

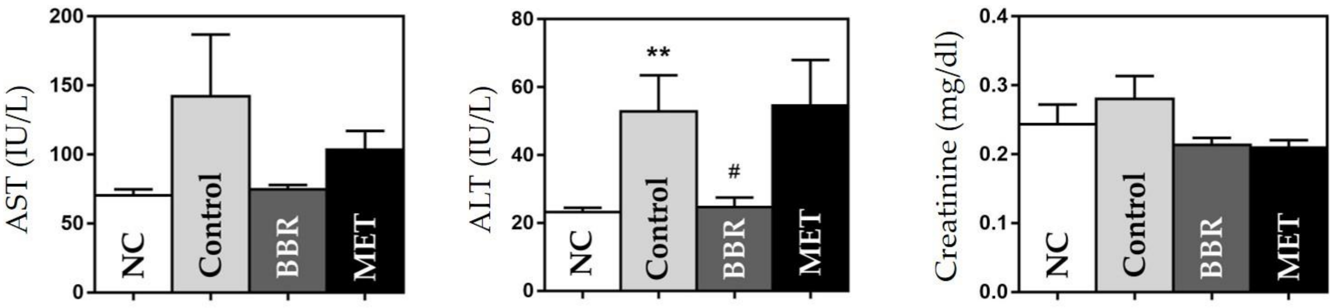

(**a**) (**b**) (**c**)

Supplementary Figure S1. Safety profile of BBR administration. **(a)** AST levels, **(b)** ALT levels, **(c)** Creatinine levels. Data are presented as mean ± standard error of the mean (SEM). **$p < 0.01$ versus the NC group and # $p < 0.05$ versus the control group.

Supplementary Tables

Supplementary Table S1. Composition of High Fat Diets (D12492).

Description	Ingredients	Grams
Fat	Lard	245.0
Fat	Soybean Oil, USP	25.0
Protein	Casein, Lactic, 30 mesh	200.0
Protein	Cystine, L	3.0
Carbohydrate	Lodex 10	125.0
Carbohydrate	Sucrose, Fine Granulated	72.8
Fiber	Solka Floc, FCC200	50.0
Mineral	Mineral mix	50.0
Vitamin	Choline Vitartrate	2.0
	Vitamin mix	1.0
		Total: 773.8

Supplementary Table S2. The primers used in reverse transcription PCR.

Gene	Primer
TNF-*α*	5′-TTCTG TCTAC TGAAC TTCGG GGTGA TCGGT CC-3′<br>5′-484 GTATG AGATA GCAAA TCGGC TGACG GTGTGGG-3′
F4/80	5′-CTTTGGC-485 TATGGGCTTCCAGTC-3′<br>5′-GCAAGGAGGACAGAGTTTATCGTG-3′
CCL2	5′-486 AGGTCCCTGTCATGCTTCTGG-3′<br>5′-CTGCTGCTGGTGATCCTCTTG-3′
CCL4	5′-487 CTCAGCCCTGATGCTTCTCAC-3′<br>5′-AGAGGGGCAGGAAATCTGAAC-3′
CCL5	5′-TGCCCACGTCAAGGAGTATTTC-3′<br>5′-AACCCACTTCTTCTCTGGGTTG-3′
CXCR4	5′-TCAGTGGCTGACCTCCTCTT-3′<br>5′-CTTGGCCTTTGACTGTTGGT-3′
GAPDH	5'-AG-TCCATGCCATCACTGCCACC-3′<br>5'-CCAGTGAGCTTCCCGTTCAGC-3'
